# Exploiting nonionic surfactants to enhance fatty alcohol production in *Rhodosporidium toruloides*

**DOI:** 10.1002/bit.27285

**Published:** 2020-02-11

**Authors:** Di Liu, Gina M. Geiselman, Samuel Coradetti, Ya‐Fang Cheng, James Kirby, Jan‐Philip Prahl, Oslo Jacobson, Eric R. Sundstrom, Deepti Tanjore, Jeffrey M. Skerker, John Gladden

**Affiliations:** ^1^ Department of Biomass Science and Conversion Technology Sandia National Laboratories Livermore California; ^2^ Department of Energy Agile BioFoundry Emeryville California; ^3^ QB3‐Berkeley University of California Berkeley California; ^4^ Biological Systems and Engineering Division Lawrence Berkeley National Laboratory Berkeley California; ^5^ Advanced Biofuels and Bioproducts Process Development Unit Lawrence Berkeley National Laboratory Emeryville California; ^6^ Joint BioEnergy Institute Emeryville California

**Keywords:** fatty alcohol, nonionic surfactants, product export, *R. toruloides*

## Abstract

Fatty alcohols (FOHs) are important feedstocks in the chemical industry to produce detergents, cosmetics, and lubricants. Microbial production of FOHs has become an attractive alternative to production in plants and animals due to growing energy demands and environmental concerns. However, inhibition of cell growth caused by intracellular FOH accumulation is one major issue that limits FOH titers in microbial hosts. In addition, identification of FOH‐specific exporters remains a challenge and previous studies towards this end are limited. To alleviate the toxicity issue, we exploited nonionic surfactants to promote the export of FOHs in *Rhodosporidium toruloides*, an oleaginous yeast that is considered an attractive next‐generation host for the production of fatty acid‐derived chemicals. Our results showed FOH export efficiency was dramatically improved and the growth inhibition was alleviated in the presence of small amounts of tergitol and other surfactants. As a result, FOH titers increase by 4.3‐fold at bench scale to 352.6 mg/L. With further process optimization in a 2‐L bioreactor, the titer was further increased to 1.6 g/L. The method we show here can potentially be applied to other microbial hosts and may facilitate the commercialization of microbial FOH production.

## Graphical abstract

Microbial production of fatty alcohols has gained broad interests, however one major challenge for commercialization of this technology is inhibition of cell growth caused by intracellular fatty alcohol accumulation. This study develops a novel strategy that exploits nonionic surfactants to promote the export of fatty alcohols from *R. toruloides*, an oleaginous yeast that is considered an attractive host for fatty acid‐derived chemicals. The method significantly alleviated growth inhibition and enhanced fatty alcohol titers and yields.

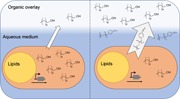

## 1 INTRODUCTION

The global energy crisis and environmental concerns surrounding nonrenewable energy production have increased the interest in alternative sustainable routes to the production of renewable fuels and chemicals (Peralta‐Yahya, Zhang, del Cardayre, & Keasling, 2012; Yaegashi et al., 2017). At present, biodiesel, surfactants, and lubricants are mostly produced from animal fat and vegetable oils (Fillet et al., 2015). A sustainable alternative to produce these products is through engineering microbial cell factories that can directly use renewable and cost‐effective feedstocks, such as lignocellulose, atmospheric CO_2_, and syngas (Peralta‐Yahya et al., 2012). A vast number of chemicals have been successfully produced in various microbes that feature diverse chemical structures and functional groups (Borodina et al., 2015; Jiang, Qiao, Bentley, Liu, & Zhang, 2017; Lee et al., 2019; Luo, Cho, & Lee, 2019; Wehrs et al., 2019). Recent advances in metabolic engineering and synthetic biology have provided a greatly expanded set of tools necessary to assemble and optimize metabolic pathways for improved titers, productivities and yields (D. Liu, Evans, & Zhang, 2015; Y. Liu & Nielsen, 2019; Salis, Mirsky, & Voigt, 2009).

However, a major challenge for the microbial production of bulk chemicals is that the target compounds are often toxic to the cell at a high level (Dunlop, 2011). This toxicity poses a metabolic burden to the host and could lead to a stress response that may reduce cell viability and trigger mutations to deactivate the pathway, thereby decreasing titers (Wu et al., 2016). Furthermore, cells that acquire mutations deactivating the pathway often exhibit growth advantages and thus will gradually dominate the population. Such heterogeneity will lead to a further decrease in product titer and yield (Xiao, Bowen, Liu, & Zhang, 2016).

To address this issue, researchers have explored various methods to improve product tolerance, including engineering efflux pumps, overexpressing stress response proteins, and modifying membrane fluidity (Dunlop, 2011; Y. Hu, Zhu, Nielsen, & Siewers, 2018). To improve biofuel tolerance in *Escherichia coli*, Dunlop et al. (2011) screened through a number of efflux pumps identified from various bacterial species. The beneficial pump significantly alleviated the toxicity and improved biofuel production. Alternatively, as a more general approach that does not distinguish biofuel specificities, overexpression of heat shock proteins has proved to be effective to improve biofuel tolerance and yields in *Clostridium acetobutylicum* (Tomas, Welker, & Papoutsakis, 2003) and *E. coli* (Reyes, Almario, & Kao, 2011). These technologies are often effective, however, they require genetic modifications that might cause an additional burden to the cell or interfere with cell physiology (Dunlop, Keasling, & Mukhopadhyay, 2010; Wagner et al., 2007). As a result, enhanced product tolerance does not necessarily guarantee higher yields (Atsumi et al., 2010; Zhao et al., 2003). Furthermore, efflux pumps that are specific to the target products are often unknown, especially in nonmodel organisms, therefore limiting the applicability of such technologies (Y. Hu et al., 2018). Another approach noninvasive to cells' genotype is improving product recovery technologies. Addition of organic overlays has proved to be effective to improve the recovery rates of various products, including isobutanol, fatty acid ethyl esters, terpenes, and so on (Alonso‐Gutierrez et al., 2013; Steen et al., 2010; Varman, Xiao, Pakrasi, & Tang, 2013). While these methods significantly enhanced product export, the efficiency of such methods vary depending on the nature of the interactions between the product, cell membrane, the supernatant, and the organic layer. The products unable to exit the cell may hinder cell growth or inhibit the bioproduct biosynthetic pathway (Zhou et al., 2016). For example, hydrophobic compounds tend to intercalate into the cell membrane, which can alter the membrane integrity and ultimately lead to cell death (Dunlop, 2011). Thus, methods to enhance the efficiency of product removal from the cell are needed to enhance production.

*Rhodosporidium toruloides* (also known as *Rhodotorula toruloides*) is an oleaginous yeast that has been regarded as a potential industrial host to produce lipid‐based bioproducts (Zhu et al., 2012), such as fatty alcohols (FOHs), which are widely used in the chemical industry as detergents, lubricants, and cosmetic additives (Fillet et al., 2015; Zhou, Buijs, Siewers, & Nielsen, 2014). However, FOHs are known to be toxic to various microorganisms, including *E. coli*, *Saccharomyces cerevisiae* and *Yarrowia lipolytica* (C. Wang, Pfleger, & Kim, 2017; W. Wang et al., 2016; Zhou et al., 2016). In this study, we exploited nonionic surfactants to promote the efficient exit of FOH from *R. toruloides* cells. All of the surfactants tested exhibited enhanced FOH titers, and tergitol NP‐40 was found to be the most effective at the lowest concentration. Subsequent analysis showed that the alleviation of toxicity significantly enhanced cell growth and shifted the population distribution towards high FOH producing cells. Overall, the use of nonionic surfactants presents an attractive approach for efficient bioproduct removal from microbial cell factories.

## 2 MATERIALS AND METHODS

### 2.1 Plasmids and strains

Codon optimization, gene synthesis, and plasmid construction were performed by Genscript (Piscataway, NJ). The genes encoding fatty alcohol reductases (FARs) were codon optimized for *R. toruloides* based on a custom IFO0880 codon usage table. The FARs were cloned under the control of a GAPDH promoter (JBEI registry: JPUB_013267; nourseothricin resistance) or an ANT1 promoter (JBEI registry: JPUB_013283; hygromycin resistance) and inserted into plasmid pGI2 (Nora et al., 2019). The FAR expression cassettes were then introduced into *R. toruloides* via *Agrobacterium tumefaciens‐*mediated transformation (ATMT). For each construct, 48 colonies were screened for FOH production and the highest producer was identified and used in this study. In the library of the strains we screened, a high FOH producing strain expressing a FAR from *Marinobacter aquaeolei* under the control of the GAPDH promoter was denoted as maquFOH. A low fatty alcohol (LFOH) producing strain expressing a FAR from domestic goose under the control of the ANT promoter was denoted as LFOH. Strains and plasmids used in this study can be found on the Agile BioFoundry Registry and are available upon request (http://public-registry.agilebiofoundry.org/; Ham et al., 2012).

### 2.2 Medium and culture conditions

Synthetic defined (SD) medium was made with 1.7 g/L yeast nitrogen base (743‐31278; BD Biosciences), 0.79 g/L complete supplement mix (1001‐010; Sunrise Science), and 40 g/L glucose. The pH was adjusted to 7.0 with NaOH. Strains were first cultivated overnight in SD medium (200 rpm, 30°C), and the overnight SD cultures were inoculated into a fresh SD medium with a starting optical density (OD) of 0.025. Then, the overnight cultures were inoculated into a fresh SD medium with a starting OD of 0.1, and 20% vol/vol dodecane (D221104; Sigma) and various detergents were added as specified. The cells were cultured for 5 days for further analysis.

### 2.3 Growth of mixed population in the presence of tergitol

To measure the population distribution of high and low FOH producing strains in the presence of tergitol, maquFOH and LFOH strains were cultured as follows. The overnight culture of maquFOH and LFOH were inoculated to a fresh SD medium with an initial OD of 0.025. The overnight cultures were then mixed together in a ratio of equal amounts (inoculation OD = 0.1 for each culture) into SD medium with 20% dodecane overlay, with and without 0.1% tergitol. The mixed cultures were cultivated for 5 days and were serially diluted for plating onto antibiotic plates.

### 2.4 Cell growth assay

Cell growth curves were recorded on an infinite F200PRO (TECAN) plate reader. Strains were first cultivated overnight in SD medium with 4% glucose. The overnight SD cultures were inoculated into a fresh SD medium with an initial OD of 0.025. The overnight cultures were then inoculated into a fresh SD medium with an initial OD of 0.1 with 20% dodecane, with and without 0.1% tergitol as specified. Relative cell density (in arbitrary units) was measured by monitoring absorption at 600 nm. Data were taken every 1,000 s for 25 hr.

### 2.5 FOH quantification

To measure FOH production, 100 μl of 1‐tridecanol C13:0 (T57630; Sigma; 1 g/L in dodecane) were added to the cultures as an internal standard. Then 60 μl of the dodecane overlay was sampled and diluted into 300 μl ethyl acetate (1007891000; Sigma). FOHs were quantified by gas chromatography with a flame ionization detector. For each sample, the column was equilibrated at 150°C for 3 min, followed by a ramp to 245°C at 20°C/min, and then was held at this temperature for 6 min. Final FOH concentrations were measured by comparing the peak areas of cetyl alcohol C16:0, palmitoleyl alcohol C16:1, 1‐heptadecanol C17:0, stearyl alcohol C18:0, and oleyl alcohol C18:1 to that of the C13:0 internal standard with calibration curves from a custom FOH standard mix in ethyl acetate.

### 2.6 Bioreactor fermentation

FOH production in *R. toruloides* was examined in a 2 L bioreactor (BIOSTAT B, Sartorius, Germany). Fermentation process parameters were controlled with temperature at 30°C, agitation at 400 rpm, and airflow at 0.5 VVM (0.37 LPM). The initial pH was adjusted to 6.0 using phosphate buffer and left uncontrolled during the run. Foaming was controlled by addition of Antifoam 204 as needed. 750 ml SD medium (50 g/L glucose, 6.7 g/L YNB without amino acids, 0.79 g/L CSM, 100 mM phosphate, 100 μM iron sulfate, with and without 0.1% tergitol) was inoculated with an overnight culture of strain maquFOH at an initial OD of 0.1. One fifty milliliters of dodecane was added as an overlay with 200 mg/L pentadecane as an internal standard. Broth samples (∼5 ml) were collected at a series of time points to measure cell density, FOH titer, glucose concentration, and nitrogen level. At each sampling point, glucose levels were restored to 50 g/L using a 600 g/L glucose solution.

## 3 RESULTS

**Figure 1:**
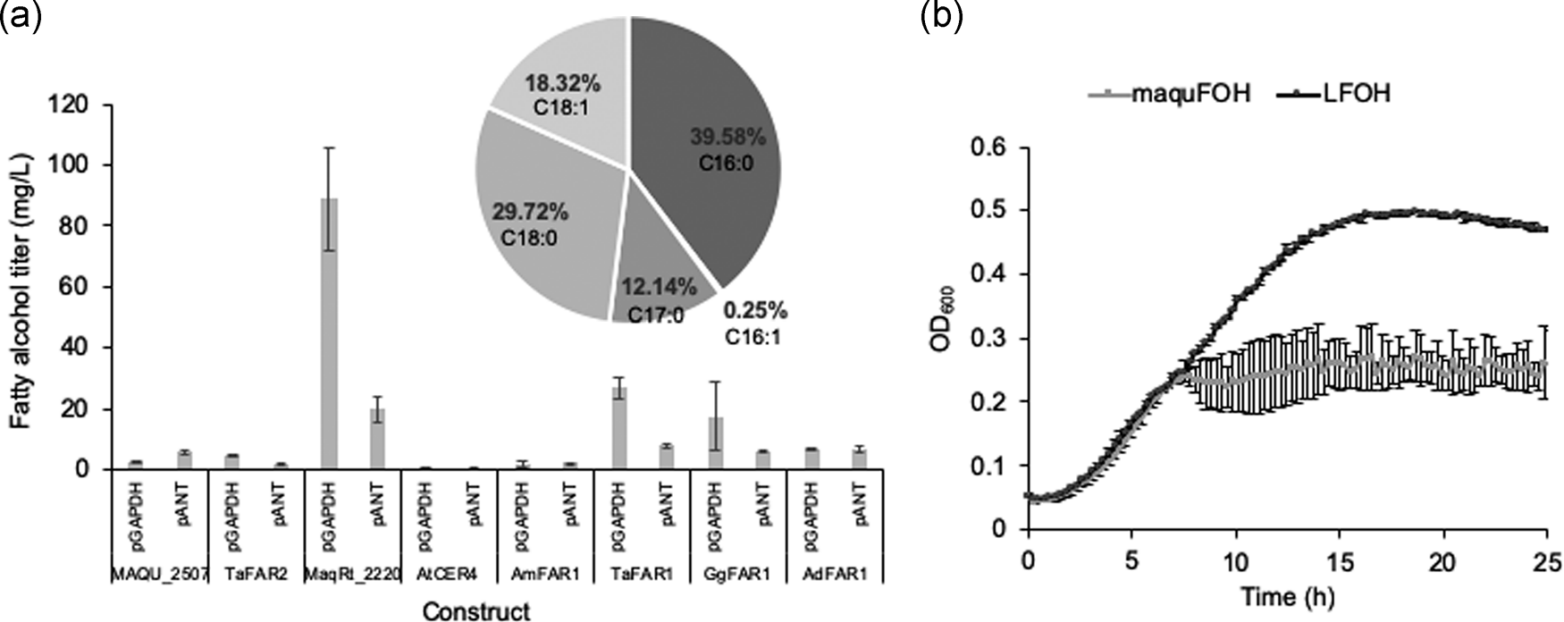
Characterization of fatty alcohol production and cell growth in *Rhodosporidium toruloides*. (a) Fatty alcohol production from various fatty acyl‐CoA reductases under two promoters, pGAPDH and pANT. The chain length distribution of fatty alcohols are shown in the inset. (b) Cell growth of a high fatty alcohol‐producing strain (maquFOH) and a low fatty alcohol producing strain (LFOH)

To construct a FOH producing pathway, 8 fatty‐CoA reductases (FARs) were identified from various organisms and were codon‐optimized for expression in *R. toruloides* IFO0880 (Table 1) using a custom codon usage table, then placed under the control of two native promoters pANT and pGAPDH (see Section 2). These genes were introduced into *R. toruloides* via ATMT and were then screened for FOH production. The titer of the highest producer from each construct was selected. Among them, a strain expressing a FAR from *M. aquaeolei* under control of the GAPDH promoter (pGAPDH‐maqu2220) produced the highest amount of FOHs, reaching 88.9 mg/L (Figure 1a). This strain is then denoted as maquFOH. The FOH composition in this strain was 39.6% C16:0, 0.3% C16:1, 12.1% C17:0, 29.7% C18:0, and 18.3% C18:1.

**Table 1:** Source organisms of fatty acyl‐CoA reductases used in this study

Gene	Source Organism	Reference
Maqu2507	*Marinobacter aquaeolei*	Fillet et al. (2015)
TaFAR2	Barn owl	Hellenbrand, Biester, Gruber, Hamberg, and Frentzen (2011)
Maqu2220	*Marinobacter aquaeolei*	Fillet et al. (2015)
AtCER4	Arabidopsis	Rowland et al. (2006)
AmFAR1	Honey bee	Teerawanichpan, Robertson, and Qiu (2010)
TaFAR1	Barn owl	Hellenbrand et al. (2011)
GgFAR1	Domestic chicken	Hellenbrand et al. (2011)
AdFAR1	Domestic goose	Hellenbrand et al., (2011)

**Figure 2:**
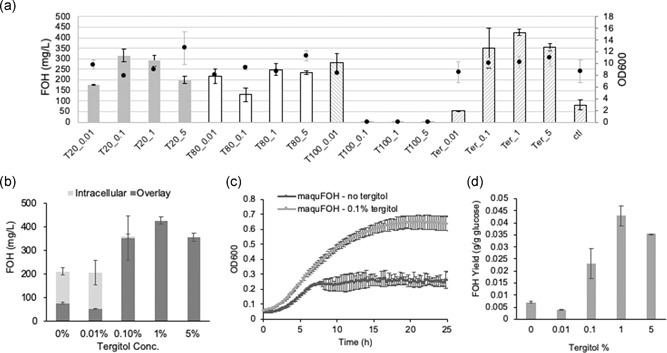
Effects of detergents on fatty alcohol titer, cell growth, and fatty alcohol yield. (a) Fatty alcohol production (bars) and cell growth (dots) of maquFOH under a gradient of various detergents. T20, T80, T100 and Ter denote TWEEN 20, TWEEN 80, Triton‐X100, and tergitol; 0.01, 0.1, 1, and 5 denote the percentage of the respective detergents. (b) Fatty alcohol concentrations in the overlay and inside the cell. (c) Cell growth of maquFOH with 0.1% tergitol and without tergitol. (d) Fatty alcohol yields under various tergitol concentrations

We compared the growth of this strain to a low FOH producing strain that expresses AdFAR (pANT‐AdFAR), denoted as LFOH. These two strains grew at a similar rate for the first 7 hr, after which the maquFOH strain reached the stationary phase whereas the LFOH strain continued to grow to a higher OD (Figure 1b). We hypothesized that the toxicity was caused by intracellular FOH accumulation, as growth inhibition caused by FOH has been observed in a few different organisms. To test our hypothesis, we measured the intracellular FOH in the maquFOH strain, which reached 134.3 mg/L. The extracellular FOH titer in this strain was 1.7‐fold lower, indicating that the majority of the FOH was trapped inside the cell (Figure 2b). This indicates that dodecane overlay alone is not sufficient to extract the majority of the FOH out of the cell. Due to the hydrophobic nature of long‐chain FOHs, this observation is not surprising.

To engineer *R. toruloides* towards high‐titer production of FOH, it is critical to address the FOH toxicity issue and develop methods to enable efficient removal of them from the cells. In *S. cerevisiae*, it has been found in a number of cases that addition of detergents can help facilitate export of hydrophobic compounds, such as farnesol, pigments, and androsta‐1,4‐diene‐3,17‐dione (Z. Hu, Zhang, Wu, Qi, & Wang, 2012; Muramatsu, Ohto, Obata, Sakuradani, & Shimizu, 2008; Z. Wang, Zhao, Hao, Chen, & Li, 2004). Therefore, we hypothesized that detergents may also improve the removal of FOH from *R. toruloides* cells.

To determine whether nonionic surfactants could facilitate the export of FOH, we tested several surfactants, including TWEEN 20, TWEEN 80, Triton‐X100, and tergitol, and supplemented 0.01%, 0.1%, 1%, and 5% of each detergent to the medium. FOH titers were measured after culturing for 5 days. A significant increase in FOH titers up to 5.2‐fold was observed for TWEEN 20, TWEEN 80, and tergitol at almost all conditions tested (Figure 2a). Triton‐X100 at concentrations higher than 0.1% was toxic to the cell and resulted in no cell growth. Across all other conditions, no significant difference in the final cell density was observed. To understand whether the detergents helped to facilitate product export, we then picked tergitol as a model surfactant to analyze intracellular and extracellular FOH levels. Large amounts of intracellular FOH (1.7‐fold of extracellular FOH) were accumulated without tergitol, whereas little intracellular FOH was accumulated for tergitol concentrations higher than 0.1%. Furthermore, the total FOH titers at elevated tergitol levels were also higher than the lower tergitol levels, indicating that lower intracellular FOH levels promoted FOH production. This is likely due to the alleviation of FOH toxicity caused by more efficient FOH extraction from the cell. Indeed, strains growing under 0.1% tergitol showed significantly improved growth compared with without tergitol (Figure 2b). With tergitol, the strains started off with a slightly higher growth rate and reached a much higher final OD at 25 hr. As a result of alleviated toxicity, the FOH yields were significantly improved to as high as sixfold, possibly due to less carbon and energy spent on stress response (Figure 2d). Interestingly, the yield under 1% tergitol was almost twofold as high as that under 0.1% tergitol; however, the FOH titers and final ODs under these two conditions were similar (within 1.2‐fold). As a small amount of intracellular FOH accumulates under 0.1% tergitol and no detectable intracellular FOH under 1% tergitol, we reasoned that even a small amount of intracellular FOHs may cause cell stress that diverts cellular resources away from FOH production, therefore leading to decreased product yields.

**Figure 3:**
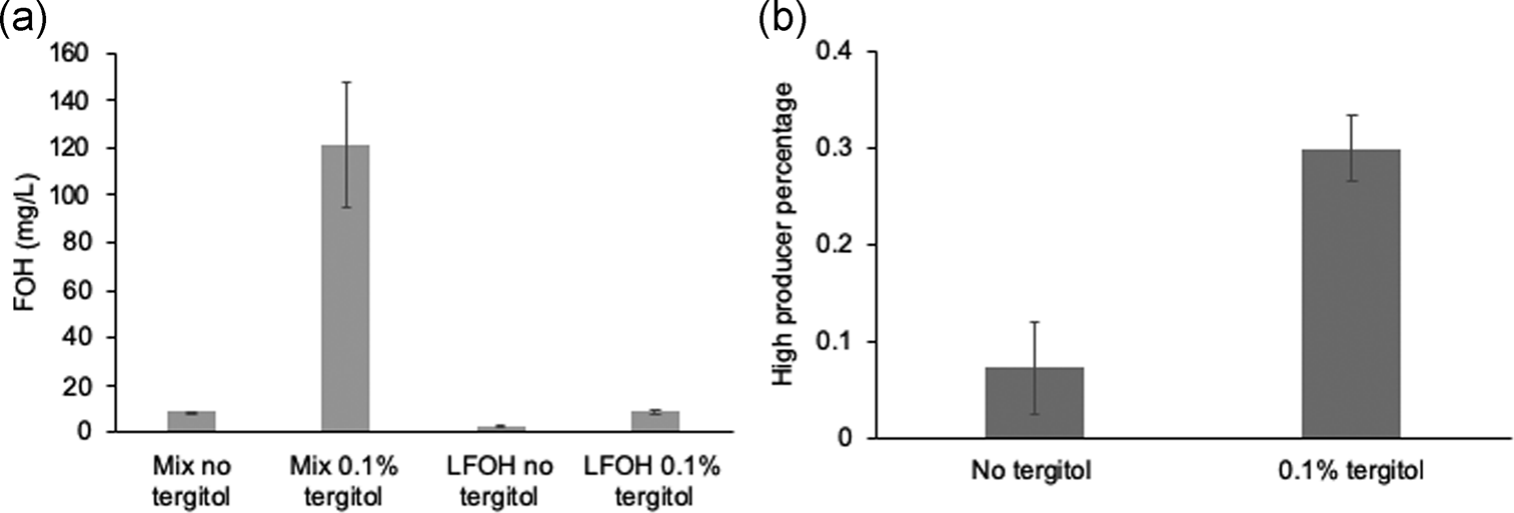
Fatty alcohol production and high producer percentage in the mixed culture of maquFOH and LFOH. (a) Fatty alcohol titers for LFOH and the mixed culture with and without tergitol. (b) The population percentage of maquFOH in the mixed culture with and without tergitol

To further verify the mechanism of how tergitol improves FOH production, we set up a growth assay to test the effects of tergitol on population distribution. An isogenic population is known to exhibit high variability in protein and metabolite production. Such variations originate from naturally inherent factors such as uneven cell division, stochastic gene expression and protein activities (Lidstrom & Konopka, 2010; Müller, Harms, & Bley, 2010; Taniguchi et al., 2010). Engineering approaches to improve the fitness of high producers are highly desirable to enhance product titers (Xiao et al., 2016). We hypothesize that the addition of tergitol will alleviate product toxicity for high producers, therefore enriching the high producer population. To test our hypothesis, we set up a growth competition assay using maquFOH and LFOH strains. A pure culture of each strain was mixed in equal amounts (OD = 0.1) both with and without tergitol, then grown for 5 days. FOH titers were measured and the cultures were plated on antibiotic plates that will select for either the maquFOH or LFOH strain for cell counting (colony forming units or CFUs). The mixed culture of maquFOH and LFOH produced 121.7 mg/L FOH with 0.1% tergitol, which is 13‐fold higher than without tergitol addition (8.6 mg/L; Figure 3a). As a control, the LFOH titer increased from 2.8 to 8.5 mg/L with the addition of tergitol, indicating LFOH contributes minimally to the titer in the mixed culture with tergitol (Figure 3a). As expected, in the mixed culture without tergitol the faster growing LFOH strain dominates the population and maquFOH only accounts for 7% of the population (Figure 3b). When 0.1% tergitol was added, the growth inhibition of the maquFOH strain was alleviated as demonstrated by its significantly improved relative fitness, reaching 30% of the population (Figure 3b). The dramatic increase in maquFOH population suggests that tergitol shifts the population distribution favorably for the higher producers, but does not completely equalize growth rates between the two strains. In addition, since the high producers do not exhibit growth advantage against low producers, the method cannot be directly used as a selection method for high producers from a mutant library. However, it can facilitate isolation of high FOH producing strains during engineering efforts and may be more useful as strains are engineered to produce industrially relevant FOH titers where toxicity could be extreme without tergitol.

**Figure 4:**
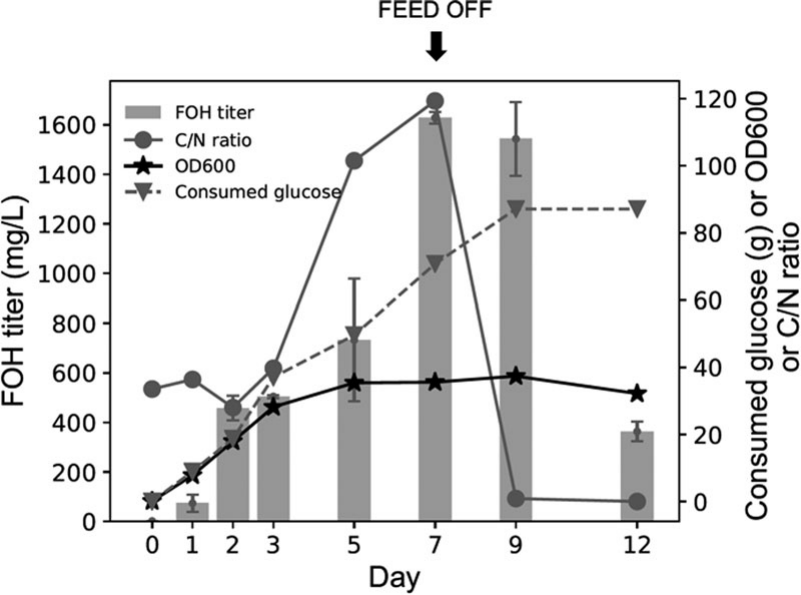
The fatty alcohol production profile of maquFOH in a 2 L fed‐batch bioreactor with the addition of 0.1% tergitol. Fatty alcohol concentrations, cell growth, glucose concentrations, and C/N ratio are plotted against time

Finally, we assessed the performance of maquFOH in a 2 L glucose fed‐batch fermenter. After 7 days of cultivation, the culture reached an OD_600_ of 35.6 and the FOH titer reached 1.6 g/L (Figure 4). In the first 3 days, the strain grew rapidly and reached 75.8% of the maximum cell density. During this time, FOH only accumulated to 31.0% of the peak FOH titer. Strikingly, more than 50% of the FOH was accumulated between Day 5 and Day 7 whereas cell density remained almost the same during this time. It was interesting to note that the C/N ratio was dramatically higher during this time, which was mainly driven by a decrease in nitrogen level (Figure 4). Low levels of nitrogen are known to induce lipid accumulation in this and other organisms (Bellou, Triantaphyllidou, Mizerakis, & Aggelis, 2016; Coradetti et al., 2018). Thus, the higher FOH productivity during this period was likely caused by a higher flux towards the fatty acid pathway induced by elevated C/N ratios. Interestingly, the FOH titer dropped significantly after the feed was turned off and glucose was fully consumed on Day 9. In fact, 76.5% of FOH was consumed within 3 days, which indicates *R. toruloides* has the ability to efficiently catabolize FOH, at least in the absence of glucose. Thus, it will be critical to identify and knock out FOH degradation pathways to improve the yield of FOH. A control 2 L glucose fed‐batch bioreactor cultivation without tergitol was also run and results were consistent with the small‐scale cultivations. After 7 days of cultivation, the culture reached an OD_600_ of 19.1 and a FOH titer of 0.65 g/L, which is 1.9‐fold and 2.5‐fold, respectively lower than the 0.1% tergitol cultivation (Figure S1). Further optimization of fermentation conditions (such as C/N ratio, pH, stir rate, etc.) and strain genotype might further improve the titer and yield of FOH.

## 4 DISCUSSION

FOHs have diverse applications in the chemical industry and have a global market size of $5.2 billion in 2011 with projections to grow at 4% CAGR in the following decade (Fillet et al., 2015). Two major challenges for successful commercialization of FOH production are low cell viability caused by product toxicity and complicated yet expensive extraction processes (Y. Hu et al., 2018). To overcome these limits, we evaluated the effects of various surfactants on the export of FOH in *R. toruloides* and found a significant increase in FOH export, which led to the titer to increase from 81.8 to 352.6 mg/L in test tubes with 0.1% tergitol. With further process optimization in a 2 L fed‐batch fermenter, the FOH titer was further increased to 1.6 g/L. This study demonstrates the utility of nonionic detergents in promoting FOH export, which could potentially be used as a tool to advance engineering this organism towards industrially relevant titers, rates, and yields. Once more advanced high FOH TRY strains are developed in future studies, a careful techno‐economic analysis comparing the costs and yield gains for a range of different detergents will be necessary to determine their viability in industrial‐scale applications.

An alternative and complementary approach to prevent intracellular FOH accumulation would be to focus on engineering transport proteins that can promote greater export of FOH out of the cell. In the past, hydrophobic molecules were believed to cross membranes by simple diffusion, and more evidence now has shown that protein transporters facilitate the transport of many hydrophobic molecules such as triglycerides, fatty acids and alkanes (Claus, Jezierska, & Van Bogaert, 2019). It has been suggested that transporters in the resistance‐nodulation‐division family and ATP‐binding cassette (ABC) family offer promises to promote the export of biofuel compounds in gram‐negative bacteria (Doshi, Nguyen, & Chang, 2013; Minty et al., 2011). However, transporters that are specific to high‐value products such as FOHs are rarely reported in eukaryotes, even in the model organism *S. cerevisiae* (Y. Hu et al., 2018). The identification and experimental validation of these transporters remain challenging due to difficulties in producing and purifying membrane proteins to obtain their crystal structures and complexities in setting up assays to perform transport studies on hydrophobic substrates. In addition, the expression of membrane proteins themselves is often toxic to the host. Thus, it remains challenging to engineer membrane proteins to facilitate hydrophobic product export in eukaryotes and other approaches like the one described here may be more fruitful in the near term.

The export of lipophilic compounds relies on a few mechanisms, including altering the membrane/cell wall composition, activating excretion mechanisms based on ABC transporters, and forming extracellular membrane vesicles for secretion (Claus et al., 2019). Naturally, some yeasts secrete surfactants to promote the accessibility of hydrophobic substrates to the cell membrane (Cirigliano & Carman, 1984; Hua, Chen, Lun, & Wang, 2003). These surfactants can reduce the size of the hydrophobic droplets, therefore increasing the contact area of these hydrophobic substrates to the cell membrane (Mlícková et al., 2004; Thevenieau et al., 2010). Likewise, the higher FOH extraction efficiency observed in this study may be due to a more efficient contact between FOH and cell membrane in the presence of surfactants, or alternatively, more efficient contact between the FOH in the cell membrane and hydrophobic overlay used to extract them from the culture. Further work is needed to uncover the exact mechanism of action.

Overall, we have demonstrated nonionic surfactants can facilitate the extraction of FOH from *R. toruloides* cells, thereby alleviating growth inhibition caused by FOH accumulation. As a result, FOH titers, productivities and yields were significantly improved. This method can also be potentially extended to facilitate the export of other lipophilic products in various organisms.

## CONFLICT OF INTERESTS

The authors declare that there are no conflict of interests.

## Supporting information

Supporting information
