# Supplementary material for: Exploiting nonionic surfactants to enhance fatty alcohol production in Rhodosporidium toruloides
Source: Biotechnol Bioeng. 2020 Feb 11;117(5):1418–25. doi: 10.1002/bit.27285 (PMC7187362; doi:10.1002/bit.27285)
Supplement: Supplementary file 1 — Supporting information [file BIT-117-1418-s001.docx]

Supplementary Information

**Exploiting nonionic surfactants to enhance fatty alcohol production in *Rhodosporidium toruloides***

Di Liu^1,^ *, Gina M. Geiselman^1^, Samuel Coradetti^1^, Ya-Fang Cheng^2^, James Kirby^1^, Jan-Philip Prahl^3, 4^, Oslo Jacobson^3, 4^, Eric R. Sundstrom^3, 4^, Deepti Tanjore^3, 4^,Jeffrey M. Skerker^2^, John Gladden^1, 5,^ *


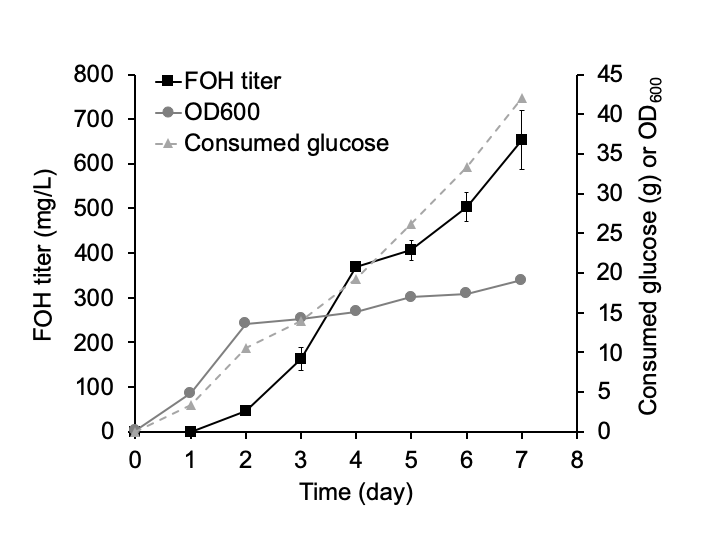


Figure S1. Fatty alcohol production profile of maquFOH in a 2 L fed-batch bioreactor without tergitol addition. Fatty alcohol concentrations, cell growth, and glucose concentrations are plotted against time.
